# Gender differences in response to an opportunistic brief intervention for obesity in primary care: Data from the BWeL trial

**DOI:** 10.1111/cob.12418

**Published:** 2020-10-07

**Authors:** Kate Tudor, Sarah Tearne, Susan A. Jebb, Amanda Lewis, Peymane Adab, Rachna Begh, Kate Jolly, Amanda Daley, Amanda Farley, Deborah Lycett, Alecia Nickless, Paul Aveyard

**Affiliations:** ^1^ Nuffield Department of Primary Care Health Sciences University of Oxford Oxford UK; ^2^ NIHR Oxford Biomedical Research Centre Oxford University Hospitals Oxford UK; ^3^ Institute of Applied Health Research University of Birmingham Birmingham UK; ^4^ Population Health Sciences, Bristol Medical School University of Bristol Bristol UK; ^5^ School of Sport, Exercise, and Health Sciences Loughborough University Loughborough UK; ^6^ Faculty Research Centre for Advances in Behavioural Science Coventry University Coventry UK; ^7^ School of Chemistry University of Bristol Bristol UK

**Keywords:** brief opportunistic intervention, gender, primary care, randomized controlled trial, weight loss

## Abstract

Weight loss programmes appeal mainly to women, prompting calls for gender‐specific programmes. In the United Kingdom, general practitioners (GPs) refer nine times as many women as men to community weight loss programmes. GPs endorsement and offering programmes systematically could reduce this imbalance. In this trial, consecutively attending patients in primary care with obesity were invited and 1882 were enrolled and randomized to one of two opportunistic 30‐second interventions to support weight loss given by GPs in consultations unrelated to weight. In the support arm, clinicians endorsed and offered referral to a weight loss programme and, in the advice arm, advised that weight loss would improve health. Generalized linear mixed effects models examined whether gender moderated the intervention. Men took effective weight loss action less often in both arms (support: 41.6% vs 60.7%; advice: 12.1% vs 18.3%; odds ratio (OR) = 0.38, 95% confidence interval (CI), 0.27, 0.52, *P* < .001) but there was no evidence that the relative effect differed by gender (interaction *P* = .32). In the support arm, men accepted referral and attended referral less often, 69.3% vs 82.4%; OR = 0.48, 95% CI, 0.35, 0.66, *P* < .001 and 30.4% vs 47.6%; OR = 0.48, 95% CI, 0.36, 0.63, *P* < .001, respectively. Nevertheless, the gender balance in attending weight loss programmes closed to 1.6:1. Men and women attended the same number of sessions (9.7 vs 9.1 sessions, *P* = .16) and there was no evidence weight loss differed by gender (6.05 kg men vs 4.37 kg women, *P* = .39). Clinician‐delivered opportunistic 30‐second interventions benefits men and women equally and reduce most of the gender imbalance in attending weight loss programmes.

**What is already known about this subject?**

- Structured community weight loss programmes are more effective in achieving weight loss than unguided weight loss attempts.
- Such programmes are used overwhelming by women, with one in 20 users being men. Even when referred by GPs, 1 in 10 are men. Community weight loss programmes present themselves as highly feminized.
- Men express preferences for weight loss support that runs differently and it is assumed that men's behaviour and preferences are immutable and that weight loss programmes tailored to men are required.

**What this study adds**

- In this trial, GPs opportunistically offered weight loss support, including referral to community weight loss programmes to unselected patients with obesity attending for routine medical care.
- Seven in 10 men accepted a referral and were nearly as likely as women to do so. Three in 10 men compared with 5 in 10 women attended the programme. This uptake closed the gender gap in referrals from 9:1 to 1.6:1.
- When GPs endorse and offer referral, this removes most of the gender gap in uptake of weight management support.

## 1 BACKGROUND

The prevalence of obesity in men and women is similar^1^ as is the proportion of men and women with obesity who are trying to lose weight.^2^ However, findings from high‐income countries indicate that men are less likely to attend weight loss programmes.^3^, ^4^, ^5^ For example, in an audit of 1.3 m people attending a weight loss programme in the United Kingdom, only 5% of users were men.^3^ This matters because self‐directed weight loss attempts are less successful than supported attempts.^6^ Men are also less likely to receive treatment for obesity in routine clinical practice^7^, ^8^ and are underrepresented in clinical trials of weight loss interventions.^5^, ^9^, ^10^

This gender gap has attracted considerable attention. In 2014, a series of systematic reviews examined the effect of gender on the clinical effectiveness and cost‐effectiveness of interventions to treat obesity.^5^ These found that men expressed a preference for fact‐based advice, delivered in social settings and programmes with a greater emphasis on physical activity. This has led to efforts to develop programmes with specific appeal to men, notably the Football Fans in Training (FFIT) scheme. Here a 12‐week programme was developed for men and delivered in football/soccer clubs, which led to a mean weight loss of 5.6 kg at 1 year compared with 0.6 kg weight loss among participants in a waiting list control group.^11^ However, the reviews also showed that men who attended mixed gender programmes were successful in losing weight. Indeed, once enrolled, men were less likely than women to drop out of programmes than women and lost relatively more weight.^5^ Surprisingly, there has been little attention given to attracting more men to existing mixed gender community weight loss group programmes that are already known to be effective.^12^, ^13^, ^14^, ^15^ This approach is likely to be less costly and quicker to implement than developing, evaluating, and scaling up novel gender‐specific schemes since it could use the established population scale infrastructure for weight management.

In routine clinical practice in the United Kingdom, only 10% of primary care referrals to community weight loss programmes are for men.^4^, ^16^ However, this in itself does not show whether clinicians do not offer referrals to men or whether men decline it. In the current pre‐planned exploratory study, we examine how men react to an opportunistic face‐to‐face brief intervention by a primary care clinician compared with women and whether this closes the gender gap in engagement in a weight loss programme.

## 2 METHODS

### 2.1 Study design and participants

The trial was approved by the NHS Research Ethics Service and registered prospectively ISRCTN: 26563137. The protocol and the primary outcome have been published previously.^17^, ^18^ The study was a parallel, two‐arm randomized trial of a brief intervention for the treatment of obesity. Researchers screened consecutively attending patients at general practices in England. Patients who were identified as having obesity using ethnic specific cut‐offs were invited to participate.^19^ If they agreed they were screened for eligibility. People with limited English, people who were already attending or had attended a structured weight loss programme in the last 3 months, or women who were or were intending to become pregnant were excluded.

At the end of the consultation, clinicians randomly delivered one of two opportunistic brief interventions to all eligible participants. In the “support” arm, clinicians endorsed, offered, and facilitated a referral to one of two commercially delivered 12‐week weight loss programmes, which were offered free of charge, as in the English NHS. The number of people who accepted the referral was recorded and they were given an appointment before leaving the practice. Both programmes are well known in the United Kingdom and are advertised in the community, mainly to women who represent 95% of attendees.^3^ In the “advice” (control) arm, clinicians advised participants to lose weight to benefit their health but did not offer referral to a weight loss programme. Both the support and advice interventions were designed to be delivered in 30 seconds. The trial showed that when clinicians opportunistically endorse, offer, and facilitate a referral of unselected patients with obesity to a community weight loss programme, this was well received and led to greater weight loss at 1 year than when clinicians advised weight loss alone.^17^, ^18^

At 3 and 12 months we assessed the actions that people had taken to lose weight. We defined taking “some action” as any self‐directed effort to control diet or increase activity. We defined taking “effective action” as following a total or partial meal‐replacement weight loss programme, taking or list at, or attending a weight loss programme at either three or 12 months since there is evidence that each of these approaches is more effective than self‐directed action.^6^ We assessed the number of attendances using data from Slimming World, the weight loss programme chosen by 94% of participants who accepted a referral in the support arm. Weight was measured in light clothing at baseline and 12 months.

### 2.2 Outcomes and statistical analyses

For all analyses, we used generalized linear mixed effects models. The link function was either a logistic term for binary outcomes or identity function for continuous outcomes. Participant randomization was stratified by general practice, so this was added as a random effect for all analyses. Analyses were conducted in SPSS version 23.

#### 2.2.1 Did trial enrolment differ by gender?

Of those invited to participate, we examined whether men were more or less likely to be enrolled. The outcome variable was trial enrolment and the denominator was all those screened with an eligible body mass index (BMI) and body fat percentage. We examined whether any differences between genders could be explained by the prevalence of exclusion criteria (eg, pregnancy, recently or currently attending a weight loss programme).

#### 2.2.2 Did gender moderate the effect of the intervention on weight loss attempts, use of effective aids to weight loss, or weight loss?

We examined whether gender moderated the effectiveness of the intervention in promoting action to lose weight. The model to do so included baseline weight, trial arm, and the interaction between gender and trial arm. The denominator was all enrolled participants. For the first analysis, the outcome variable was any reported action to lose weight (ie, self‐directed efforts or effective action). For the second analysis, the outcome variable was people specifically reporting taking effective action to manage their weight.

We also examined whether the effect of trial arm on weight loss differed by gender; including baseline weight, trial arm, gender, and the interaction between gender and trial arm. The outcome variable was weight at 12 months. We weighed 1419 (75%) participants at 12 months. Otherwise, we imputed missing weights at 12 months using the baseline observation carried forward (BOCF) approach.

#### 2.2.3 Did men and women differ in the response to clinicians' brief interventions?

In the support arm only, we examined whether gender was associated with accepting the clinician's referral to a weight loss programme (ie, telling the clinician that they would attend). The denominator was everybody in the support arm. We also examined whether men differed from women in the likelihood of attending the programme at least once, which amounts to acting on the clinician's recommendation. We assessed attendance among all those randomized to the support arm and among those who accepted the referral. To examine whether the programme was acceptable to those who experienced it at least once, we examined whether mean number of attendances at the programme differed by gender, with the denominator being all those who attended at least once.

## 3 RESULTS

### 3.1 Did trial enrolment differ by gender?

Between 4 June 2013 and 23 December 2014, 2730 people with obesity were offered enrolment in the trial. Of these, 1637 were women and 1064 were men. Data for gender was missing for 29 potentially eligible patients and these people were excluded from further analyses. A greater proportion of men who were potentially eligible were enrolled compared with women (75.8% vs 65.7%, odds ratio (OR) = 1.63, 95% confidence interval (CI), 1.36, 1.95, *P* < .001). This was because a smaller proportion of men were not eligible to participate compared with women (3.9% vs 13.3%, OR = 0.35, 95% CI, 0.26, 0.48, *P* < .001), primarily because men were less likely to be currently or recently participating in a weight loss programme, although pregnancy or intended pregnancy also excluded some women (Table 1). Clinicians deemed it inappropriate to make an opportunistic brief intervention to the same proportion of men and women (4.5%, OR = 1.00, 95% CI, 0.71, 1.39, *P* = .99). There was no evidence of gender differences in the proportion of people who declined to participate (16% vs 17%, OR = 0.93, 95% CI 0.75, 1.15, *P* = .50).

**TABLE 1:** Proportion of potentially eligible patients enrolling in the trial by gender

	Men n	Women n	Missing gender n	OR (95% CI)^a^	Sig
Eligible BMI/invited	1064	1637	29	–	–
Declined participation (%)	167 (15.7)	271 (16.6)	18	0.93 (0.75, 1.15)	0.50
Not eligible for other reasons (%)	41 (3.9)	218 (13.3)	3	0.35 (0.26, 0.48)	<0.001
Pregnant	0 (0)	34 (2.1)	0	–	–
Participating in weight loss programme	14 (<1)	58 (3.5)	1	–	–
Participated in weight loss programme in past 3 mo	13 (1.2)	64 (3.9)	1	–	–
Visiting clinician for weight loss	5 (<1)	21 (1.3)	0	–	–
Poor English language skills	3 (<1)	5 (<1)	0	–	–
Clinician deemed brief intervention participation inappropriate (%)	48 (4.5)	74 (4.5)	0	1.00 (0.71, 1.39)	0.99
Enrolled (%)	806 (75.8)	1076 (65.7)	0	1.63 (1.36, 1.95)	<0.001

Abbreviation: BMI, body mass index.

^a^ Men compared with women (reference).

### 3.2 Did gender moderate the effect of the intervention on weight loss attempts, use of effective aids to weight loss, or weight loss?

Data on actions to lose weight were available for 1560 participants (657 men and 903 women) (Table 2). The clinician's offer of support increased the proportion of people taking action to manage their weight from 83.0% in the advice arm to 88.9% OR = 1.62, 95% CI, 1.21, 2.16, *P* = .001 but there was no evidence overall that the proportion of men and women taking action differed (OR = 0.75, 95% CI, 0.47, 1.20, *P* = .23). There was also no evidence that men and women differed in their response to the support intervention; 87.7% of men and 89.6% of women in the support arm took action compared with 83.0% of men and 83.1% of women in the advice arm (*P* = .60 for the interaction).

**TABLE 2:** Self‐reported actions and weight loss for men and women in the advice and support arm

	Advice only men (N = 340)	Advice only women (N = 460)	Advice total (N = 800)	Support men (N = 317)	Support women (N = 443)	Support total (N = 760)
Effective action	41 (12.1)	84 (18.3)	125 (15.6)	132 (41.6)	269(60.7)	401 (52.8)
Some action	241 (70.9)	298 (64.8)	539 (67.4)	146 (46.1)	128 (28.9)	274 (36.1)
No action	58 (17.1)	78 (17.0)	136 (17.0)	39 (12.3)	46 (10.4)	85 (11.2%)
12‐mo weight loss (BOCF) (kg)	0.68 (5.71)	1.32 (5.33)	1.04 (5.50)	2.39 (6.71)	2.46 (6.32)	2.43 (6.49)

Altogether, 52.8% of participants in the support arm took effective action to manage their weight compared with 15.6% in the advice arm (OR = 6.12, 95% CI, 4.82, 7.78, *P* < .001). For the main effects of the model, men were less likely than women to take effective action in both support and advice arms, with 41.6% of men and 60.7% of women taking effective action in the support arm and 12.1% of men and 18.3% of women in the advice arm doing so (OR = 0.38, 95% CI, 0.27, 0.52, *P* < .001). There was no evidence that the relative effect of the support arm differed by gender (*P* = .32 for the interaction).

At 12 months, we collected weight data for 1419 participants (73% of men and 77% of women) and weight loss was 2.43 kg (6.49) in the support arm and 1.04 kg (5.50) in the advice arm. In the support arm, weight loss in men was 2.39 kg (6.71) compared with 2.46 kg (6.32) in women. In the advice arm, weight loss in men was 0.68 kg (5.71) compared with 1.32 kg (5.33) in women. There was no evidence that gender moderated the relationship between intervention group and weight loss (*P* = .26 for the interaction).

### 3.3 Did men and women differ in the response to clinicians' brief interventions?

There were 401 men and 539 women assigned to the support arm and were offered a referral to a weight loss programme. Men were less likely than women to accept the referral (69.3% vs 82.4%; OR = 0.48, 95% CI, 0.35, 0.66, *P* < .001) (Table 3).

**TABLE 3:** Proportion of men and women accepting and attending the referral and weight loss in the support arm

	Men	Women	OR (95% CI)	Sig
Total randomized to support arm, n	401	539	–	–
Accepted referral, n (%)	278 (69.3)	444 (82.4)	0.48 (0.35, 0.66)	<0.001
Attended weight loss programme (overall), n (%)	122 (30.4)	257 (47.6)	0.48 (0.36, 0.63)	<0.001
–	–	–	Mean difference (95% CI)	–
Number of sessions attended, mean (SD)	9.66 (3.24)	9.08 (3.47)	0.62 (−0.25, 1.48)	0.16
12‐mo weight loss BOCF in support arm (N = 940), mean (SD)	2.39 (6.71)	2.46 (6.32)	−0.53 (−1.42, 0.37)	0.25
12‐mo weight loss BOCF in participants who attended weight loss programme (N = 379), mean (SD)	6.05 (8.56)	4.37 (7.57)	0.80 (−1.01, 2.62)	0.39

Abbreviations: BOCF, baseline observation carried forward; CI, confidence interval; OR, odds ratio.

Among all those in the support arm, 379 (40.3%) people attended a weight loss programme, but men were less likely to do so than women (30.4% vs 47.6%; OR = 0.48, 95% CI, 0.36, 0.63, *P* < .001). In those accepting a referral, men were less likely to attend the programme (43.2% vs 57.9%; OR = 0.58, 95% CI, 0.43, 0.79, *P* < .001). Of those who attended the programme at least once, there was no evidence of a difference by gender in the number of sessions attended (9.7 vs 9.1; mean difference = 0.62, 95% CI, −0.25, 1.48, *P* = .16). Among this group, men lost slightly but not significantly more weight than women at 12 months; 6.05 kg (8.56) compared with 4.37 kg (7.57), mean adjusted difference = 0.80, 95% CI, −1.01, 2.62 (*P* = .39) (Table 3).

## 4 DISCUSSION

### 4.1 Abstract

More than four in five men and women opportunistically approached in a primary care clinic volunteered to enrol in a trial of brief interventions for obesity. Randomization to a clinician offering, endorsing, and facilitating a referral to a weight loss programme increased the proportion of men and women taking action to lose weight. There was no evidence of a gender difference in the effect of the intervention itself, but overall, men were less likely to take effective action to manage their weight than women. This was manifest in a greater proportion of men declining the clinician's offer of a referral and, among those who agreed to it, a smaller proportion of men attending the programme compared with women. However, once enrolled in the programme, men attended a similar number of sessions as women and there was no evidence that weight loss at 12 months differed by gender.

### 4.2 Strengths and limitations

This is the first analysis of the effect of an intervention that can be delivered by clinicians in routine practice to increase the engagement of men to established mixed gender weight loss programmes. It is based on a randomized controlled trial in which 75% of participants were weighed, which is higher than the typical follow‐up in similar weight loss trials (63%).^20^

Whilst we planned these exploratory analyses^17^ these subgroup analyses did not inform the sample size calculations. In many cases, we did not detect differences between men and women, but the trial was not planned to have sufficient power to detect these subgroup effects and the precision of the confidence intervals means that we may have missed modest but important differences in effectiveness by gender. We cannot therefore conclude that the intervention effect does not differ by gender, only that there is no evidence that it does so and that there are no moderate or large differences.

### 4.3 Comparison with existing literature

**FIGURE 1:**
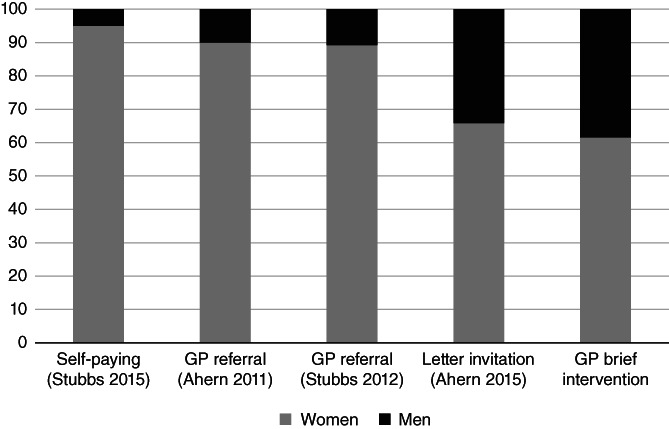
Gender balance in enrolment in weight loss programs

Five percent of paying customers of the commercial weight loss group programmes are men,^3^ whilst 10% of people referred by clinicians to these programmes are men.^4^, ^16^ Why might men be so underrepresented in such programmes? Previous findings have suggested that men view community weight loss programmes as feminized spaces.^21^, ^22^ The evidence here is that such a perception can be easily overcome by clinicians endorsing and offering referral. We asked participants to rate the acceptability of the referral to a weight loss programme immediately after the consultation, and there was no evidence that scores differed between men and women. Moreover, men's behaviour in this trial shows clear evidence of the broad acceptability of these programmes to most men. Here 39% of participants in the weight loss programme were men and the gender ratio in attendance was 1:1.6 compared with 1:10 in routine primary care. This is reinforced by data from two clinical trials where men and women were offered an equal opportunity to attend a weight loss programme by receiving an invitation letter to do so.^10^, ^13^ Around a third of people enrolled were men, a gender ratio of 1:1.9 in a trial where the gender mix of the invited population was known. As Figure 1 suggests, most of the gender imbalance in paying customers appears to be due to men's reluctance to enrol in such programmes. However, this trial suggests that the gender imbalance in routine primary care is because clinicians offer programmes mainly to women, perhaps because they perceive such programmes to be gendered. Data from this trial shows, however, that three quarters of the large gender imbalance is removed by clinicians spending 30 seconds to endorse and offer such a programme.

Our findings also indicate that when men attended at least one session of the programme, they remained as engaged as women (attending the same number of total sessions) and lost a similar amount of weight. This is consistent with evidence from a clinical trial^15^ and an audit of people who chose to self‐fund attendance at similar programmes.^3^ Once men are enrolled in these programmes, they find them appropriate and engagement does not differ by gender.

Many national guidelines recommend that clinicians refer men and women with obesity to behavioural weight loss programmes.^23^, ^24^, ^25^ However, the frequency of brief interventions and attendance at programmes is much lower than occurred in this trial,^26^, ^27^ suggesting that clinicians are not adhering to guidelines. There is evidence that clinicians do not offer referrals to weight loss programmes opportunistically because they are concerned about the best way to initiate conversations about weight loss.^28^, ^29^ Where conversations do occur, these are usually initiated by patients not clinicians.^26^ Since men are less likely to problematize their weight than women,^22^ they may raise the issue with their clinician less often, reducing the likelihood of being offered support. These results should reinforce clinicians' sense of capability at having these conversations. A relatively brief intervention with a clinician was able to overcome a preference against apparently feminine weight loss programmes that many had regarded as immutable.

### 4.4 Implications for research and practice

Currently, clinician referrals represent a small proportion of all people using community weight loss programmes; most users are self‐payers, and nearly all of them are women. Research is needed to understand how else we can counter the perception that these programmes are designed for and best suited to women. If a 30‐second intervention by a clinician can remove three quarters of the gender imbalance, this should encourage efforts to do so for self‐payers, because this perception seems easily malleable.

These results have direct application to clinical practice. It appears that clinicians mainly offer to refer women to weight loss programmes, perhaps because they share the widespread belief that these are feminized and unsuitable for men. However, clinicians can be reassured that their endorsement appears to counter that and three quarters of the gender imbalance in referrals can be removed by clinicians endorsing and offering such programmes equally to men and women. Men and women benefited markedly and equally from these brief opportunistic interventions.

Currently there is no direct evidence that gender‐specific interventions, for example those delivered in sports clubs or at work, are more effective or cost‐effective for men than existing evidence‐based programmes. The FFIT programme, specifically developed and tested for men resulted in a mean weight loss of 5.6 kg at 1 year with an intervention lasting 12 sessions and costing an estimated £680.^11^ A trial of a mixed gender community weight loss group for 12 or 52 weeks shows mean weight losses at 1 year of 4.8 and 6.8 kg costing £60 or £195, respectively.^15^ Whilst gender‐specific programmes may be useful for men who are unwilling to attend existing services, such programmes are not yet widely available. Policy might therefore focus more on implementing brief interventions as a way to reach men rather than on developing bespoke weight loss programmes for them.

## 5 CONCLUSIONS

Brief opportunistic interventions by clinicians to unselected patients with obesity to endorse, offer, and facilitate a referral to an effective weight loss programme slightly increase the proportion of men and women taking action on their weight and markedly increase the proportion taking effective action. Brief opportunistic interventions work equally effectively in men and women and remove three quarters of the gender imbalance in referrals to these programmes seen in routine care. Once enrolled, men attend and achieve as much weight loss as women do. Clinicians can use these findings to more frequently offer referrals to these programmes for men as well as women.

## CONFLICT OF INTEREST

Slimming World and Rosemary Conley donated free weight loss programmes to support the trial by covering NHS treatment costs but otherwise had no role in the study or the decision to submit the manuscript. Paul Aveyard and Susan A. Jebb are investigators on a trial funded by Cambridge Weight Plan. Paul Aveyard has done half a day of consultancy for Weight Watchers. Amanda Farley reports grants from Ethicon (Johnson and Johnson) outside the submitted work. Kate Jolly reports grants from National Prevention Research Initiative of the UK, administered by the MRC during the conduct of the study. Deborah Lycett reports grants from MRC NPRI, during the conduct of the study; and Deborah Lycett received donations of referral vouchers from Slimming World to the NHS for a study that ran from 2011 to 2015; this provided no financial benefit to her or her employer. All other authors declare no competing interests bar the involvement of Slimming World and Rosemary Conley in the trial. None of the investigators received personal payments for these relationships.

## AUTHORS' CONTRIBUTIONS

Susan A. Jebb, Amanda Lewis, Sarah Tearne, Paul Aveyard, Rachna Begh, Kate Jolly, Amanda Daley, Amanda Farley, Deborah Lycett, Alecia Nickless and Peymane Adab developed the trial protocol and oversaw the trial. Sarah Tearne managed the trial. Susan A. Jebb, Amanda Lewis, Sarah Tearne, Paul Aveyard, Rachna Begh, Kate Jolly, Amanda Daley, Amanda Farley, Deborah Lycett, Alecia Nickless and Peymane Adab developed the overall analysis plan. Kate Tudor, Sarah Tearne and Peymane Adab developed the analysis plan for this analysis. All authors contributed to the drafting of the manuscript. Guarantors: Kate Tudor, Sarah Tearne and Paul Aveyard.

## ETHICS STATEMENT

The trial had approval from the NHS Research Ethics Service and is registered ISRCTN: 26563137, Registered 14 November 2012, http://www.isrctn.com/ISRCTN26563137. Informed consent was gained from all trial participants. This work uses data provided by patients and collected by the NHS as part of their care and support and would not have been possible without access to this data. The NIHR recognizes and values the role of patient data, securely accessed and stored, in both underpinning and leading to improvements in research and care.

## Data Availability

The datasets analysed during the current study are not yet publicly available because the analysis by the authors is ongoing. However, the data may be available on reasonable request from the chief investigator for the trial.
